# Siz2 Prevents Ribosomal DNA Recombination by Modulating Levels of Tof2 in Saccharomyces cerevisiae

**DOI:** 10.1128/mSphere.00713-19

**Published:** 2019-11-27

**Authors:** Neethu Maria Abraham, Kathirvel Ramalingam, Saketh Murthy, Krishnaveni Mishra

**Affiliations:** a Department of Biochemistry, School of Life Sciences, University of Hyderabad, Hyderabad, India; University College Dublin, Belfield

**Keywords:** rDNA recombination, Siz2, Ulp2, Fob1, Tof2, SUMOylation

## Abstract

Ribosomal DNA (rDNA) recombination in budding yeast is regulated by multiple converging processes, including posttranslational modifications such as SUMOylation. In this study, we report that the absence of a SUMO E3 ligase, Siz2, results in increased unequal rDNA exchange. We show that Siz2 is enriched at the replication fork barrier (RFB) in the rDNA and also controls the homeostasis of Tof2 protein. *siz2Δ* resulted in increased accumulation of total Tof2 in the cell and a consequent increase in the enrichment of Tof2 at the rDNA. Overproducing Tof2 ectopically or conditional overexpression of Tof2 also resulted in higher levels of rDNA recombination, suggesting a direct role for Tof2. Additionally, our chromatin immunoprecipitation (ChIP) data indicate that the accumulation of Tof2 in a *siz2Δ* mutant resulted in an enhanced association of Fob1, an RFB binding protein at the rDNA at the RFB. This increased Fob1 association at the RFB may have resulted in the elevated rDNA recombination. Our study thus demonstrates that the Tof2 levels modulate recombination at the rDNA.

**IMPORTANCE** The genes that encode rRNA in Saccharomyces cerevisiae are organized as multiple repeats. The repetitive nature and heavy transcription of this region make it prone to DNA breaks. DNA breaks could lead to recombination, which could result in either loss or gain of repeats with detrimental consequences to the cell. Multiple mechanisms operate to maintain the stability of rDNA. Earlier studies reported that the absence of Ulp2, a deSUMOylase, resulted in declining levels of Tof2 and thereby disrupted rDNA silencing. In contrast, our findings suggest that accumulation of Tof2 can also result in increased rDNA recombination, through a mechanism that involves Fob1, an RFB-bound protein. While our study has examined only Tof2, rDNA recombination could be regulated by other proteins through a mechanism similar to this.

## Abstract (precis)

The genes that encode rRNA in Saccharomyces cerevisiae are organized as multiple repeats. The repetitive nature and heavy transcription of this region make it prone to DNA breaks. DNA breaks could lead to recombination, which could result in either loss or gain of repeats with detrimental consequences to the cell. Multiple mechanisms operate to maintain the stability of rDNA. Earlier studies reported that the absence of Ulp2, a deSUMOylase, resulted in declining levels of Tof2 and thereby disrupted rDNA silencing. In contrast, our findings suggest that accumulation of Tof2 can also result in increased rDNA recombination, through a mechanism that involves Fob1, an RFB-bound protein. While our study has examined only Tof2, rDNA recombination could be regulated by other proteins through a mechanism similar to this.

## INTRODUCTION

**FIG 1:**
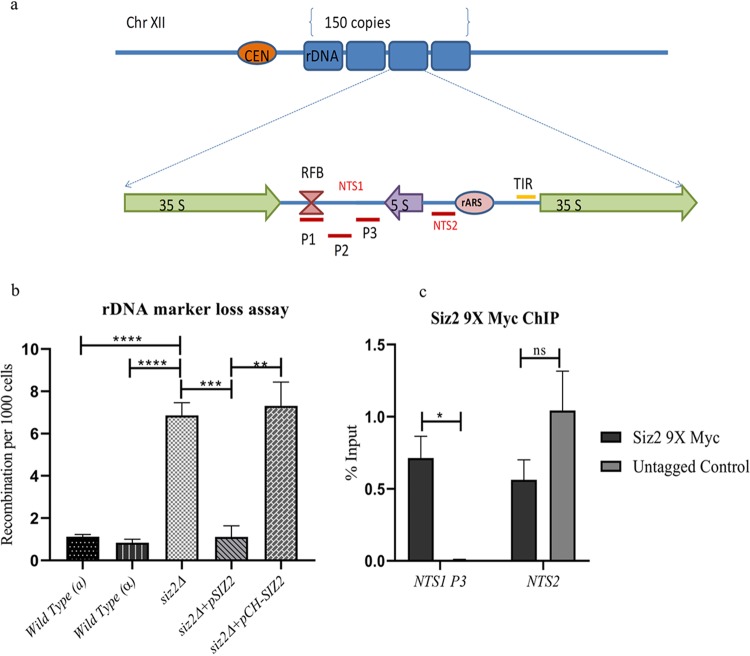
*siz2Δ* causes increased rDNA USCE. (a) Schematic representation of rDNA array and location of primer regions tested by ChIP to detect enrichment of proteins (P1 to P3) for *NTS1* and *NTS2*. (b) Recombination marker assay was carried out in wild-type *mat***a** (KRY 486), wild-type α (KRY 2129), and *siz2Δ* (KRY 1821) strains; KRY 1821 transformed with *pSIZ2* (CKM 230); and a catalytically inactive mutant of Siz2, *pCH-SIZ2* (CKM 330), by growing them in selective medium, plating them onto Sc-Ade or Sc-Leu Ade containing minimal adenine, and incubating them for 2 to 3 days. Loss of *ADE2* placed at the rDNA locus results in the accumulation of a red pigment. The number of half-sectors is then counted and represented as recombination frequency per 1,000 cells. The graph represents an average of the recombination frequency calculated for a total of 5,000 to 20,000 cells from 3 or more independent colonies, and error bars represent SEM. (c) ChIP was performed using anti-Myc antibodies in KRY 1671 (Siz2 9×Myc) and KRY 486 (no tag). The levels of enrichment at *NTS1* (P3) and *NTS2* were calculated and plotted as percent input. The graph represents an average for five experiments, and error bars represent SEM. ns, *P* > 0.05; *, *P* ≤ 0.05;  **, *P* ≤ 0.01; ***, *P* ≤ 0.001; ****, *P* ≤ 0.0001.

In most eukaryotes, the genes encoding rRNA are present as repetitive sequences clustered in one or more chromosomes. The ribosomal DNA (rDNA) of Saccharomyces cerevisiae is encoded on chromosome XII and consists of 100 to 200 copies of a 9.1-kb repeat that encodes the 5S and 35S rRNA components of the ribosome. The coding sequences are separated by two nontranscribed regions termed *NTS1* and *NTS2* (1). The 35S rRNA is transcribed by RNA polymerase I, whereas the 5S rRNA is transcribed by RNA polymerase III (2). The intergenic spacer *NTS2* contains the origin of replication (the ribosomal autonomous replicating sequence [rARS]) and cohesin-associated region (3), while *NTS1* contains a replication fork barrier (RFB) (4) and a 520-bp RNA polymerase II-dependent bidirectional promoter, E-pro (5). The transcriptions of 35S rRNA and 5S rRNA proceed in opposite directions (Fig. 1a).

During S phase, as replication proceeds bidirectionally from multiple rARS, one of the replication forks moves in the direction opposite 35S rRNA transcription and can thus encounter the transcription machinery of 35S rRNA, leading to collision between the replication and transcription complexes (4). Such collisions can result in DNA breaks which would promote recombination and chromosome instability. To prevent a collision between the transcription and replication machineries, a replication fork barrier (RFB) is present at the *NTS1* region (4). The RFB contains a 100-bp sequence of DNA that permits movement of the replication fork in the direction of 35S rRNA transcription but not in the opposite direction. This region bound by a replication fork block protein, Fob1, ensures that replication moving in the opposite direction of 35S rRNA transcription is stalled, while the forks proceeding in the direction of 35S rRNA transcription continue (6). Failure to respond to this stalling results in a double-strand break (DSB) and requires homologous recombination to repair the breaks. When the repair is accomplished using equal sister chromatid recombination, that is, the sequence recombines with DNA on the same location of the sister chromatid, the rDNA copy number remains the same (7). Recombination with a misaligned sister chromatid leads to unequal sister chromatid exchange (USCE) that causes an increase in rDNA copy number.

The recombination events are regulated by two key mechanisms: Sir2 dependent and Sir2 independent (8, 9). Sir2 is a conserved NAD^+^-dependent histone deacetylase that is crucial for establishing silent chromatin. Sir2 predominantly localizes to the nucleolus and heterochromatic loci, namely, the telomeres and silent mating-type loci. At the rDNA, Sir2 exists in a complex with Net1 and Cdc14 (together called the RENT complex) and silences the E-pro promoter found at *NTS1*, situated near RFB. Transcription from the E-pro promoter produces a noncoding transcript that prevents the association of cohesin to the rDNA at the cohesin-associated region (CAR) (10). Under normal conditions, Sir2 represses transcription of the E-pro region, and therefore, in the absence of noncoding transcripts, cohesin is recruited. Cohesin recruitment promotes equal sister chromatid exchange and subsequent repair of DSB by homologous recombination. In the second mechanism, Fob1 initiates a hierarchal protein recruitment cascade that finally recruits cohesin and condensin that prevent USCE. First, Fob1 that binds at the RFB sequence recruits Tof2. Tof2 recruits the cohibin complex consisting of Lrs4 and Csm1; the cohibin complex recruits cohesin (9). In addition, Fob1 also interacts with and stabilizes Net1 and thus can influence the Sir2-dependent pathway as well. DNA damage due to stalled replication forks at the RFB of the rDNA is also repaired using the homologous sequence of an adjacent repeat (intrachromosomal recombination), where any repeat present between the damaged unit and the donor unit loops out and excises DNA fragments in the form of extra recombinant circles, resulting in reduction in rDNA copy number (11).

Multiple means of regulation of the rDNA copy number converge to ensure the stability of rDNA. When rDNA copy number is low, disassociation of cohesin due to E-pro-mediated transcripts results in an increase in rDNA copy number (10). When brought back to the wild-type levels, Sir2-dependent and cohibin-dependent recruitment of cohesin together maintain stable rDNA copy number (9, 10). The absence of Sir2 and the cohibin complex increases unequal sister chromatid exchange and decreases replicative life span, while deletion of Fob1 reduces intrachromatid recombination, decreases extra recombinant circle production, and extends replicative life span (9, 12–14). Thus, various processes such as transcription, cohesion, replication, and recombination performed by Sir2, the cohibin and cohesin complex, and Fob1 together regulate copy number of rDNA.

Posttranslational modifications such as phosphorylation and SUMOylation have evolved as crucial regulators for rDNA stability. For instance, Fob1 phosphorylation promotes Fob1-Fob1 interaction and oligomerization. This increases intrachromatid recombination by promoting DNA interactions between RFBs at rDNA (a mechanism termed chromosome kissing). Further phosphorylation of the C-terminal domain of Fob1 promotes loading of both RENT and Tof2 complexes at the *NTS1* (15, 16).

SUMOylation is another posttranslational modification that regulates various processes by changing protein-protein interactions, localization, or levels of target proteins through ubiquitin-mediated degradation. SUMOylation is a reversible posttranslational modification that covalently attaches a SUMO moiety to a target protein in an ATP-dependent mechanism similar to ubiquitination (17). This modification is reversed by deSUMOylases or SUMO-specific proteases which cleave the SUMO from target proteins (18). The yeast genome encodes two deSUMOylases, Ulp1 and Ulp2. Ulp1, apart from deSUMOylating targets, is also essential for maturation of SUMO (19). Ulp2 removes polySUMO chains on targets and prevents their degradation (20). Ulp1 and Ulp2 have different targets partly due to their differential cellular localization. While Ulp1 is localized to the nuclear periphery, Ulp2 is found within the nucleoplasm (21, 22). Slx5/Slx8 is a class of SUMO-targeted ubiquitin ligases, which specifically recognize polySUMOylated proteins and divert them for degradation.

Substrate specificity of SUMOylation is brought about by the SUMO E3 ligases, which catalyze the transfer of SUMO from the E2-conjugating enzyme to the lysine residue on the target protein. In budding yeast, Siz1 and Siz2 are the main SUMO E3 ligases that SUMOylate a diverse set of targets (23, 24). Zip3, another SUMO E3 ligase in yeast, is involved in the assembly of synaptonemal complex between homologous chromosomes during meiosis (25), and Mms21 is involved in DNA repair, nucleolar function, and sister chromatid recombination (26–28).

Nucleolus appears to be a major site of SUMO dynamics. For instance, in wild-type cells SUMO is seen as a diffused signal within the nucleus; in the absence of the deSUMOylase Ulp2, a bulk of SUMO (visualized by using GFP-SMT3) is enriched within the nucleolus which houses the rDNA repeats (29). A reduction in rDNA copy number is seen in a *siz1Δ siz2Δ* mutant. Overexpression of the SUMO E3 ligase, Siz2, in fact, caused an increase in rDNA copy number, while shutting off the expression of Siz2 resulted in efficient loss of this amplified rDNA (29). Proteins involved in rDNA maintenance have also been shown to be SUMOylated by the yeast SUMO E3 ligases Siz1, Siz2, and Mms21 (29–33).

Recent studies have investigated the importance of SUMOylation of a few targets in rDNA silencing (31). In an *ulp2Δ* mutant, rDNA proteins Net1, Tof2, and Fob1 are hyperSUMOylated and there is a reduced occupancy at rDNA. This reduction in enrichment is reversed in the absence of Slx5. Ulp2 is recruited at the rDNA by Csm1. *ulp2Δ* resulted in elevated Tof2 polySUMOylation and a decline in its overall abundance (34, 35). The *ulp2Δ* mutant also exhibited rDNA silencing defects, possibly due to the degradation of Tof2. Biochemical characterization of SUMOylated proteins using mass spectrometry (MS) identified Ulp1-, Ulp2-, Siz1/Siz2-, and Mms21-specific targets which included Net1, Tof2, and Cdc14 (33).

SUMO ligase and SUMO protease work antagonistically to maintain levels of proteins by controlling STUbL-mediated degradation (31). In this work, we investigated the role of the SUMO E3 ligase Siz2 in preventing USCE at the rDNA. Although previous studies report a change in rDNA copy number in the *siz1Δ siz2Δ* mutant, the mechanism through which either of these enzymes acts together or individually has not been studied. Tof2 polySUMOylation and its subsequent degradation were shown to affect rDNA silencing. However, the necessity for this homeostatic control or the consequence of Tof2 accumulation has not been investigated earlier. This study bridges the gap in understanding the role of Tof2 homeostasis in rDNA recombination. We show that Siz2 affects Tof2 protein levels and its recruitment at the RFB. Increasing Tof2 at the RFB resulted in increased Fob1 binding. Thus, Siz2 is important to maintain adequate Tof2 levels and consequently adequate Fob1 at the RFB.

## RESULTS

### SUMO ligase Siz2 is required to prevent UCSE.

In an effort to understand the importance of SUMOylation in rDNA recombination, we tested the requirement for SUMO ligase Siz2 in rDNA recombination. We monitored rDNA recombination based on the loss of an *ADE2* gene placed in the rDNA repeats (9). Colonies with half-sectors of red and white which indicate the loss of the *ADE2* marker in the first mitotic division on the plate were counted. As shown in Fig. 1b, an increased marker loss indicating elevated recombination at the rDNA is seen in the *siz2*Δ mutant. Complementation with wild-type Siz2 returned recombination to wild-type levels, whereas complementation with a catalytic mutant of Siz2 did not. These data show that SUMO ligase activity of Siz2 is required for repressing recombination at the rDNA locus.

Earlier studies have shown that Siz2 is associated with several nucleolar proteins, and localization of fluorescently tagged Siz2 suggested a nuclear localization with an additional sequestration at specific parts of the nuclear envelope (29, 36–39). Since our results suggested a role for Siz2 in rDNA recombination, we tested if Siz2 acted directly at the rDNA site or influenced rDNA recombination indirectly. We performed chromatin immunoprecipitation (ChIP) for Siz2 in strains encoding Siz2 with a C-terminal fusion to 9×Myc epitopes (40). A schematic representation of the primers used for ChIP is shown in Fig. 1a. As shown in Fig. 1c, we found that Siz2 was enriched over 2-fold at the *NTS1* region of rDNA. This further suggests that Siz2 may play a role at the rDNA locus.

### Siz2 regulates rDNA recombination via Tof2.

**FIG 2:**
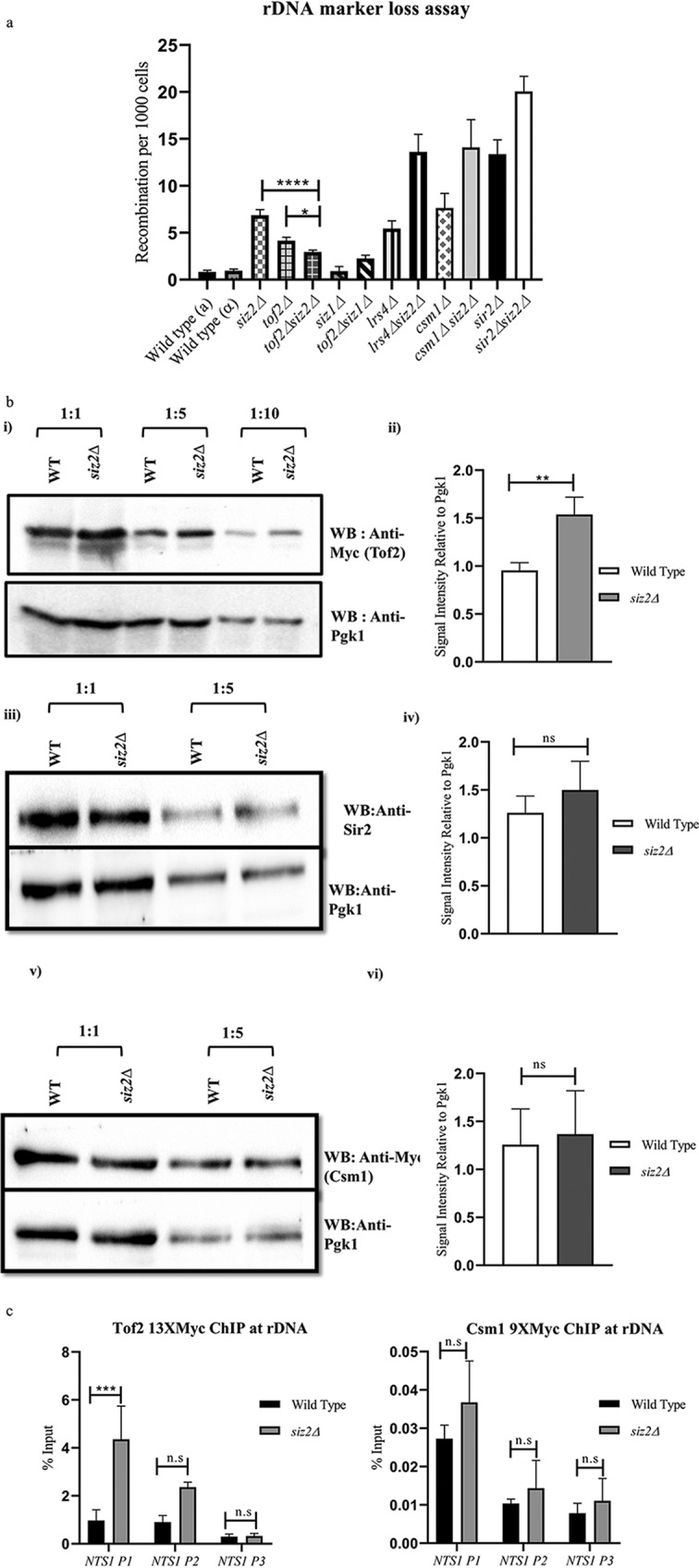
Siz2 controls rDNA recombination through Tof2. (a) Recombination frequency per 1,000 cells was measured for the following: wild-type **a** (KRY 486), wild-type α (KRY 2129), *siz2Δ* (KRY 1821), *csm1Δ* (KRY 487), *lrs4Δ* (KRY 488), *tof2Δ* (KRY 489), *sir2Δ* (KRY 1542), *sir2Δ siz2Δ* (KRY 1906), *csm1Δ siz2Δ* (KRY 1813), *lrs4Δ siz2Δ* (KRY 1816), *tof2Δ siz2Δ* (KRY 1819), *siz1Δ* (KRY 2108), and *siz1Δ tof2Δ* (KRY 2107) strains. Strains were grown in selective medium, plated onto SC-Ade plates containing minimal adenine, and incubated for 2 to 3 days. (b) Total protein was extracted from indicated strains; undiluted extract and 5-fold and 10-fold dilutions of the extracts were separated by SDS-PAGE; and Western blotting assays were performed with antibodies to Myc epitope to detect Tof2 and Csm1 or antibodies to Sir2. All blots were probed with antibodies to Pgk1 to confirm equal loading. (i) Western blot (WB) assay to detect Tof2 in wild-type (WT) and *siz2Δ* strains containing Tof2-Myc (KRY 2131 and KRY 2132). (ii) Quantification of Tof2 in KRY 2131 and KRY 2132 (*n* = 4); *n* refers to the number of Western blot assays performed using extracts from 4 independent colonies. (iii) Western blot assay to detect Sir2 in wild-type and *siz2Δ* (KRY 1923 and KRY 1924) strains. (iv) Quantification of Sir2 in KRY 1923 and KRY 1924 (*n* = 3); *n* refers to the number of Western blot assays performed using extracts from 3 independent colonies. (v) Western blot assay to detect Csm1 in wild-type and *siz2Δ* strains containing Csm1-Myc (KRY 1800 and KRY 1898). (vi) Quantification of Csm1 in KRY 1800 and KRY 1898 (*n* = 3); *n* refers to the number of Western blot assays performed using extracts from 3 independent colonies. (c) Wild-type and *siz2Δ* strains containing either 13×Myc-tagged Tof2 (KRY 1923 and KRY 1924, respectively) or 9×Myc-tagged Csm1 (KRY 1800 and KRY 1898) were used to perform ChIP with anti-Myc, and the level of enrichment at regions within the *NTS1* including the RFB (NTS1 P1) was calculated and plotted as percent input. Graph represents an average from five experiments, and error bars represent SEM. ns, *P* > 0.05; *, *P* ≤ 0.05; **, *P* ≤ 0.01; ***, *P* ≤ 0.001; ****, *P* ≤ 0.0001.

Recombination at the rDNA is regulated by both Sir2-containing RENT complex and the cohibin complex containing Tof2, Csm1, and Lrs4. As all of these proteins are potentially SUMOylated, they could be targets of Siz2 (31, 33). Therefore, we made double mutants of *siz2Δ* with each of these components and measured rDNA recombination by the half-sector assay. We found that *sir2Δ*, *lrs*4Δ, and *csm1Δ* mutants had high levels of recombination and the *tof2*Δ mutant had a lower recombination, as reported before (9). The double deletion of *siz2Δ* with each of *sir2Δ*, *lrs4Δ*, and *csm1Δ* exacerbated the recombination phenotype, suggesting that these proteins do not act in the same pathway as that of Siz2 (Fig. 2a). However, *tof2Δ siz2Δ* double mutants had lower frequencies of recombination than either of the single mutants, and thus, *tof2Δ* suppressed the *siz2Δ* phenotype. Although the *tof2Δ* mutant by itself does not have a significant recombination phenotype, it is involved in recruiting the cohibin complex which regulates UCSE as well as the binding of Fob1 and Top1, both of which are important for preventing rDNA recombination (9, 41). Thus, while the absence of cohibin complex and Sir2 exhibited additive effects with *siz2Δ*, the absence of Tof2 was able to suppress the defect seen in a *siz2*Δ mutant. We therefore reasoned that perhaps Siz2 regulates rDNA recombination through Tof2. Moreover, to confirm if the defect observed in a *siz2Δ* mutant was due to a specific function associated with Siz2, we tested if loss of *siz1*, also known to have a role in rDNA integrity, elevated recombination similarly to that of Siz2. As shown in Fig. 2a, the *siz1Δ* mutant did not exhibit the recombination phenotype and was comparable to wild type.

### Siz2 controls Tof2 protein levels.

Recent work had shown that levels of Tof2 protein decreased in the absence of Ulp2, a SUMO protease that removes polySUMO chains, and this in turn impacts silencing at the rDNA locus (34, 35). Siz2 is a known polySUMOylating enzyme, and therefore, we tested if Tof2 protein levels were affected in *siz2Δ* cells. We compared the levels of Tof2 protein in wild-type and *siz2Δ* cells and found that, indeed, Tof2 protein levels were higher in the *siz2Δ* mutant (Fig. 2b, i and ii). Under the same conditions, protein levels of Csm1 and Sir2 were not altered, further suggesting that Tof2 is a likely target for Siz2 at the rDNA (Fig. 2b, iii to vi). We next asked if this increase in Tof2 levels was also reflected in the increased association of Tof2 with the rDNA. Consistent with our observations for recombination and protein levels, Tof2 enrichment at the RFB was increased in the *siz2Δ* mutant, while Csm1 was unaffected (Fig. 2c).

### Siz2 is epistatic to Ulp2 in regulating rDNA recombination and Tof2 protein levels.

**FIG 3:**
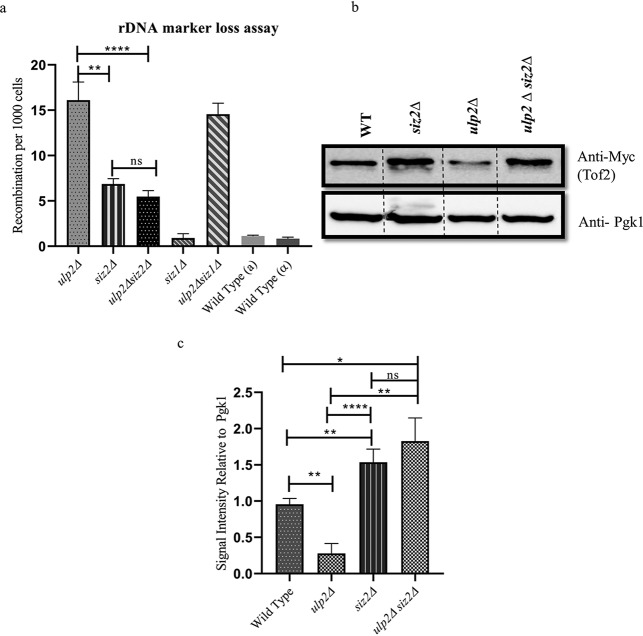
Siz2 and Ulp2 control rDNA recombination through Tof2 protein homeostasis. (a) ADE2 marker loss assay was carried out in wild-type (KRY 486, KRY 2129), *ulp2Δ* (KRY 1919), *siz2Δ* (KRY 1821), *ulp2Δ siz2Δ* (KRY 1920), *siz1Δ* (KRY 2108), and *ulp2Δ siz1Δ* (KRY 2111) strains. Strains were grown in selective medium, plated onto SC-Ade plates containing minimal adenine, and incubated for 2 to 3 days. (b) Wild-type (KRY 1923), *siz2Δ* (KRY 1924), *ulp2Δ* (KRY 1929), and *siz2Δ ulp2Δ* (KRY 1938) strains containing 13×Myc-tagged Tof2 were grown in YPD, and proteins were extracted. Protein concentration was equalized and loaded. The Western blot was developed using antibodies to Myc to detect Tof2 and Pgk1 to confirm equalized protein levels. Lanes of relevant samples from the same blot were spliced for clarity. (c) Quantification of Tof2 in the indicated strains from 3 different experiments which demonstrates the restoration of Tof2 protein levels similar to that of a *siz2Δ* mutant. ns, *P* > 0.05; *, *P* ≤ 0.05; **, *P* ≤ 0.01; ****, *P* ≤ 0.0001.

As earlier work had shown that Tof2 homeostasis depended on Ulp2, and that the *ulp2Δ* mutant exhibited a loss in silencing at the rDNA, we tested the effect of *ulp2Δ* on rDNA recombination (34). We find that loss of Ulp2 leads to elevated recombination rates, and fittingly, the *siz2 Δulp2Δ* double mutant had reduced frequency of rDNA recombination compared to that of an *ulp2Δ* mutant. The increased recombination seen in an *ulp2Δ* mutant was suppressed by the deletion of *siz2*. In fact, the recombination frequency in the *siz2Δ ulp2Δ* mutant was similar to that of the *siz2Δ* mutant (Fig. 3a). It is possible that the suppression of recombination in the *siz2Δ ulp2Δ* mutant could be independent of Tof2 levels. To test this, we introduced Tof2-Myc in the *siz2Δ ulp2Δ* double mutant and compared the total Tof2 protein levels. The recombination frequency correlated with the levels of Tof2, wherein the *ulp2Δ* mutant showed decreased levels of Tof2 as shown before (34), and *siz2Δ ulp2Δ* restored Tof2 to levels similar to those of the *siz2Δ* mutant, reflecting the recombination phenotype seen in a *siz2Δ ulp2Δ* mutant (Fig. 3b and c). Thus, *siz2Δ* is epistatic to *ulp2Δ* in regulating both Tof2 protein levels and rDNA recombination frequency. Taken together, these data indicate that Tof2 protein is maintained at a steady-state level by the opposing actions of Siz2 and Ulp2.

### Tof2 modulates recombination at rDNA.

**FIG 4:**
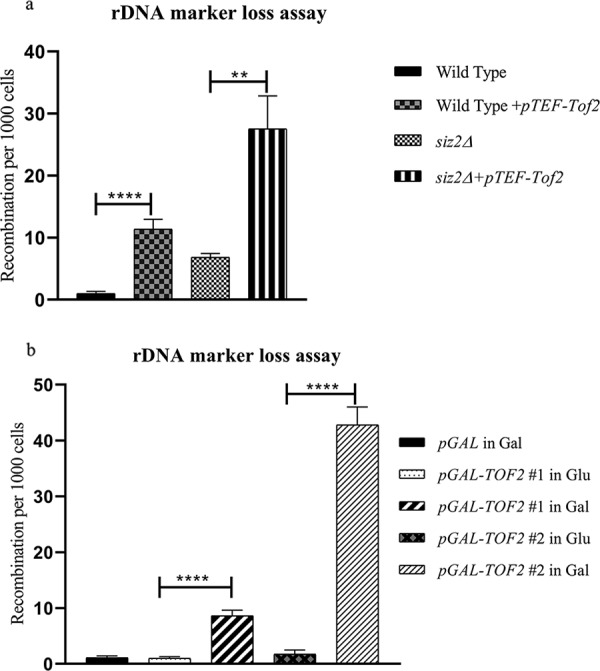
Overexpression of Tof2 elevates recombination. (a) ADE2 marker loss assays were performed in wild-type (KRY 2129) and *siz2Δ* (KRY 1821) strains transformed with *pTEF-TOF2* (CKM 610) or p*TEF* (CKM 274). Strains were grown in selective medium, serially diluted, plated onto Sc-Leu Ade containing minimal adenine, and grown at 30°C for 2 to 3 days. (b) Tof2 protein levels were checked in strains expressing different levels of Tof2 protein. KRY 1966 (#1) and KRY 1973 (#2) overexpressing Tof2 under the inducible Gal1 promoter and a control strain without Tof2 under the inducible promoter (KRY 1965) were grown in Sc-galactose or Sc-glucose medium, plated after dilution onto Sc-galactose/Sc-glucose plates containing minimal adenine, and incubated for 2 to 3 days. **, *P* ≤ 0.01; ****, *P* ≤ 0.0001.

The results described above establish that rDNA recombination is regulated by both Siz2 and Ulp2, and these proteins regulate protein levels of Tof2 (our results and reference 34). Based on these data, we hypothesized that rDNA recombination was regulated by balanced association of Tof2 at RFB. If this was indeed the case, one prediction that can be made is that overproducing Tof2, independent of perturbations to Siz2 or Ulp2, should increase recombination. Tof2 was overproduced to mimic accumulating Tof2 protein in a *siz2Δ* mutant by expressing Tof2 under a strong TEF promoter in a 2μ vector. As shown in Fig. 4a, overexpressing Tof2 in wild-type and *siz2Δ* strains increases the recombination frequency.

Two strains that express Tof2 at different levels under the inducible Gal1 promoter were created by Geil et al. (Tof2#1, moderately overexpressed; Tof2#2, highly overexpressed) (42). The same strains were crossed with an *RDN1*::*ADE2* strain to create the moderately overexpressed and highly overexpressed Tof2 containing the *ADE2* at the rDNA to test for recombination frequency. The differential protein levels in these two strains were confirmed by Western blotting (data not shown) to test for rDNA recombination. The respective strains, when grown in glucose, exhibit wild-type levels of recombination. When grown in galactose, they exhibit high recombination mimicking the *siz2Δ* mutant (Fig. 4b). The recombination phenotype was in accord with the levels of Tof2 being overexpressed, where Tof2#2 exhibited a much more severe rDNA recombination phenotype than Tof2#1.

### Tof2 potentially controls Fob1, the key recruiter of both RENT and cohibin.

**FIG 5:**
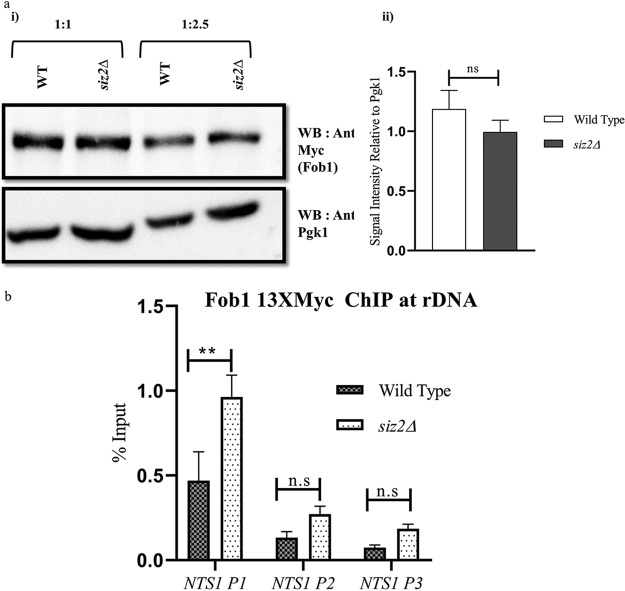
Fob1 accumulates at the RFB in the *siz2Δ* mutant. (a) (i) Wild-type and *siz2Δ* strains containing 13×Myc-tagged Fob1 (KRY 1802 and KRY 1933, respectively) were grown in YPD, and proteins were extracted. Protein concentration was equalized and loaded after diluting as indicated. Western blotting was done using antibodies (Ant) to Myc to detect Fob1 and antibodies to Pgk1 to confirm equalized protein levels. (ii) Quantification of Fob1 in KRY 1802 and KRY 1933 (*n* = 3). (b) ChIP using anti-Myc antibodies was performed in wild-type and *siz2Δ* strains containing 13×Myc-tagged Fob1 (KRY 1802 and KRY 1933, respectively). The levels of enrichment at *NTS1* and *NTS2* were calculated and plotted as percent input. The graph represents an average from six experiments, and error bars represent SEM. ns, *P* > 0.05; **, *P* ≤ 0.01.

Although decreased levels of Tof2 were earlier shown to correlate with reduced rDNA silencing, the downstream target for Tof2 is not known. We hypothesized that Fob1 could be a possible target for Tof2 based on several previous observations in literature. One, it was shown that polySUMOylated Fob1 accumulates in the *ulp2Δ* mutant and its binding was reduced by 50% in the *ulp2Δ* mutant (31). Second, although RFB is present across all 100 to 200 repeats, not all of them efficiently stall the approaching replication fork. Moreover, an approaching fork does not stall at an RFB that is not bound by Fob1, indicating that Fob1 is a major limiting factor. Third, Fob1 when overexpressed led to more efficient stalling on 10 successive RFBs placed on a minichromosome (43). Based on these observations, we first tested if Fob1 protein levels were also affected by *siz2Δ*. However, we find that, as shown in Fig. 5a (i and ii), total Fob1 protein level remains unchanged.

While Fob1 is essential for recruiting Tof2 and the cohibin complex at NTS1 (9), it was also shown that, in the absence of Tof2, Fob1 association at the RFB was reduced by half (41). Thus, an increase in Tof2 at the RFB might result in increased enrichment of Fob1 that leads to increased numbers of stalled RFBs and that induces hyperrecombination. ChIP was performed to test the Fob1 enrichment in wild-type and *siz2Δ* strains. As shown in Fig. 5b, the enrichment of Fob1 at the RFB was higher in the *siz2Δ* mutant than in the wild type. Thus, loss of *siz2* does not affect Fob1 protein level; instead, possibly through Tof2, *siz2Δ* increases Fob1 levels at the RFB.

## DISCUSSION

Studies have so far established the antagonistic actions of Ulp2 and Slx5 in protein homeostasis of rDNA-bound proteins. Our findings suggest that a similar opposing action of Siz2 and Ulp2 also regulates the homeostasis of Tof2, an rDNA-bound protein, and hence rDNA recombination.

Absence of Siz2 led to higher recombination frequency at rDNA (Fig. 1b). We tested the possible pathways through which Siz2 might act, by creating double mutants of *siz2Δ* with *sir2Δ*, *lrs*4Δ, *csm1Δ*, and *tof2Δ*. We show that only *tof2Δ* suppressed the recombination phenotype seen in a *siz2Δ* mutant, indicating that the *siz2Δ*-induced recombination requires Tof2 (Fig. 2a). Earlier studies reported that Tof2 is SUMOylated and that polySUMOylated Tof2 accumulated in the *ulp2*Δ mutant, which in turn is targeted for STUbL-mediated degradation (31, 34). Thus, while existing evidence has established the detrimental role of reduced Tof2 for rDNA, this work was able to establish that it is, in fact, a balance of Tof2 levels that is essential to prevent rDNA recombination.

We hypothesized that Siz2 possibly polySUMOylates Tof2 and that lack of polySUMOylation in the absence of Siz2 leads to accumulation of Tof2, as it cannot be diverted for degradation. Indeed, the *siz2Δ* mutant exhibited increased levels of Tof2 (Fig. 2b, i and ii). This accumulation of Tof2 also caused an enrichment of Tof2 at the RFB (Fig. 2c) within the *NTS1* region of the rDNA, which is a hot spot for recombination. Ulp2, by removing the polySUMO chains, protects Tof2 from degradation and maintains the cohibin complex at the RFB. The complementary role of Siz2 and Ulp2 in Tof2 homeostasis was established in this study when the recombination phenotype in an *ulp2Δ* mutant was suppressed in the *siz2Δ ulp2Δ* mutant, which correlated with the changes in protein levels as well (Fig. 3a to c). Thus, the effect of *ulp2Δ* on UCSE at the rDNA through Tof2 protein levels is dependent on Siz2 function.

To confirm if this accumulation was the sole contributing factor for rDNA recombination, Tof2 was overproduced by cloning it into a high-expression vector and by placing it under an inducible Gal1 promoter (Fig. 4a and b). Increased expression of Tof2 under both conditions exhibited increased USCE even in a wild-type strain. In fact, overexpressing Tof2 at different protein levels using the inducible Gal promoter led to an equivalent increase in rDNA recombination. The amount of Tof2 was directly proportional to the severity of rDNA recombination phenotype. PolySUMOylated Tof2 is usually targeted for degradation by the Slx5/Slx8-mediated ubiquitin pathway. The excessive degradation is then prevented by Ulp2 that removes polySUMO chains. This ensures that there is sufficient Tof2 to recruit the cohibin complex and thereby prevent rDNA recombination. We speculate that the roles of Ulp2 and Slx5/Slx8 at the rDNA in Tof2 homeostasis are possibly dependent on Siz2 activity.

Further, to understand the molecular mechanism by which increased accumulation of Tof2 at *NTS1* causes increased USCE, possible targets were examined. Tof2 is important for localization of the cohibin complex (Csm1/Lrs4), Top1 cleavage complex, and Fob1 (9, 41). The increased USCE observed with increased levels of Tof2 bound at the rDNA is possibly due to increased accumulation of one of these proteins. Increased accumulation of Tof2 did not affect Csm1 enrichment at the RFB region of rDNA, ruling out the possibility that the cohibin complex is the direct target of Tof2 that led to rDNA recombination.

We reasoned that Fob1 could be the downstream effector for the Tof2-induced elevated recombination in a *siz2Δ* mutant due to its dual roles in maintaining rDNA recombination. Fob1 prevents rDNA recombination by recruiting the RENT complex and cohibin complex, thus allowing only equal sister chromatid recombination (9). On the other hand, Fob1 is also the initiating factor for rDNA recombination, since it is the stalling of RFB induced by Fob1 and Fob1-mediated chromosome kissing that induces intrachromosomal hyperrecombination (15). Absence of Tof2 led to a more than 50% decrease in Fob1 binding at the RFB (41). We demonstrate that while Fob1 protein levels remain unchanged, Fob1 protein at the NTS1 was enriched (Fig. 5a and b). This enrichment could cause an increase in DSBs at the rDNA and, hence, cause increased USCE. This is possibly why not all RFBs are occupied by Fob1. This study was able to uncover a direct link between Tof2 abundance and Fob1 association at RFB to prevent USCE at the rDNA.

Our data indicate that action of Siz2 and Ulp2 maintains a balanced level of Tof2 protein. This protein homeostasis is important, and shifting the balance in either direction is deleterious. For instance, excessive degradation of Tof2 and Net1 brought about by polySUMOylation and STUbl-mediated degradation causes inefficient binding of both Net1, which recruits the RENT complex, and Tof2, which recruits the cohibin complex at the rDNA, thus increasing USCE (31). Similarly, in *siz2Δ* cells, accumulation of Tof2 and its subsequent enrichment at the RFB are potentially due to lack of Siz2-mediated polySUMOylation. We speculate that the balanced levels of Tof2 prevent excessive recruitment of Fob1 at the RFBs to maintain genome stability at the rDNA. This work provides insight into why maintenance of Tof2 level is critical: too much or too little changes the Fob1 association at RFB, thus modulating recombination, and suggests a dual role for Tof2 in preventing rDNA recombination.

## MATERIALS AND METHODS

### Strains and plasmids.

Strains and plasmids used in the study are listed in Tables 1 and 2, respectively. All knockouts described here are full-length open reading frame replacements unless otherwise stated. To construct Tof2 in p425-TEF (CKM 610), Tof2 open reading frame (ORF) from CKM 609 was digested using enzymes SpeI and XhoI and was subcloned into CKM 274. Standard yeast manipulation methods were followed. Yeast strains were grown in yeast extract-peptone-dextrose (YPD) or selection medium at 30°C.

**TABLE 1:** List of strains used in this study^*a*^

Strain no.	Genotype	Source
KRY 2	*MAT***a** W3031a	Rod Rothstein (44)
KRY 3	*MAT*α W3031b	Rod Rothstein (44)
KRY 486	KRY 2 except *RDN1*::*ADE2 RAD5*	Angelika Amon
KRY 487	KRY 486 except *csm1*Δ::*KAN Mx*	Angelika Amon
KRY 488	KRY 486 except *lrs4*Δ::*KAN Mx*	Angelika Amon
KRY 489	KRY 486 except *tof2*Δ::*KAN Mx*	Angelika Amon
KRY 1542	KRY 486 except *sir2*Δ::*KAN Mx*	This study
KRY 1671	W1588-*4C SIZ2-9*×*MYC*::*TRP1 MAT*α	X. Zhao (40)
KRY 1800	KRY 2 except *CSM1 9*×*MYC*::*TRP1*	Angelika Amon (46)
KRY 1802	KRY 3 except *FOB1 13*×*MYC*::*HIS3*	Angelika Amon (46)
KRY 1813	KRY 486 except *csm1Δ*::*KAN Mx siz2*Δ::*HIS3*	This study
KRY 1816	KRY 2129 except *lrs4Δ*::*KAN Mx siz2*Δ::*HIS3*	This study
KRY 1819	KRY 2129 except *tof2Δ*::*KAN Mx siz2*Δ::*HIS3*	This study
KRY 1821	KRY 2129 except *siz2Δ*::*HIS3 mat*α	This study
KRY 1898	KRY 2 except *CSM1 9*×*MYC*::*TRP1 siz2*Δ::*HIS3*	This study
KRY 1906	KRY 2129 except *siz2Δ*::*HIS3 sir2*Δ::*KAN Mx*	This study
KRY 1919	KRY 2129 except *ulp2Δ*::*HIS3*	This study
KRY 1920	KRY 486 except *ulp2Δ*::*HIS3 siz2*Δ::*KAN Mx*	This study
KRY 1929	KRY 3 except *ulp2Δ*::*HIS3 TOF2 13*×*MYC*::*KAN TEL VIIL*::*URA3*	This study
KRY 1923	KRY 2 except *TOF2 MYC*::*KAN Mx ADE2*	This study
KRY 1924	KRY 3 except *TOF2 13*×*MYC*::*KAN Mx siz2*Δ::*HIS3*	This study
KRY 1933	KRY 3 except *FOB1 13*×*MYC*::*HIS3 siz2*Δ::*HIS3*	This study
KRY 1938	KRY 3 except *ulp2Δ*::*HIS3 siz2Δ*::*KAN MX TOF2 13*×*MYC*::*KAN MX*	This study
KRY 1965	KRY 2129 except *LEU2*::*pGAL1-Tcyc1-LEU2 rad5-535*	This study
KRY 1966	KRY 2129 except *LEU2*::*pGAL1-TOF2 13*×*MYC-Tcyc1-LEU2* (#1, moderately overexpressed)	This study
KRY 1973	KRY 2129 except *LEU2*::*pGAL1-TOF2 13*×*MYC-Tcyc1-LEU2 rad5-535* (#2, highly overexpressed)	This study
KRY 2107	KRY 486 except *siz1Δ*::*HIS3 tof2Δ*::*KAN Mx*	This study
KRY 2108	KRY 486 except *siz1Δ*::*HIS3*	This study
KRY 2111	KRY 2129 except *ulp2Δ*::*HIS3 siz1Δ*::*HIS3*	This study
KRY 2129	KRY 486 except *MAT*α	This study
KRY 2131	KRY 3 except *TOF2 13*×*MYC*::*KAN Mx*	This study
KRY 2132	KRY 3 except *TOF2 13*×*MYC*::*KAN Mx siz2*Δ::*HIS3*	This study

a All strains used in the study were isogenic with W303**a** (*leu2-3*,*112 his3-11*,*15 URA3-1 ade2-1 trp1-1 can1-100 rad5-535*) or W1588-*4C*, a RAD5 derivative of W303.

**TABLE 2:** List of plasmids used in this study

Plasmid no.	Description	Reference or source
CKM 230	p*SIZ2* in yCpLac111	47
CKM 330	pCH-*SIZ2* in yEp Lac181	47
CKM 274	p425-*TEF*	48
CKM 609	p*GAL1*-*TOF2*-*tCYC1-LEU2*	W. Seufert (42)
CKM 610	p*TEF-TOF2*	This study

### rDNA recombination assay.

Recombination at rDNA was determined by measuring the loss of the *ADE2* gene inserted at a single rDNA at the rDNA loci (9). Loss of *ADE2* leads to the accumulation of a red pigment causing the cells/colony to become either pink or red. This method uses this phenomenon for a color-based sector assay. During plating at the time of cell division, loss of *ADE2* placed at the rDNA due to a recombination event results in a colony that is half red and half white. Such half-sectored colonies are scored as a single recombination event. The recombination frequency is calculated by counting the total number of colonies and the number of half-sectored colonies and plotted as number of half-sectors per 1,000 cells. Completely red colonies are excluded for the estimation of recombination frequency. A total of 5,000 to 20,000 colonies from 3 or more independent colonies for each genotype were counted. Strains were grown in appropriate medium and plated onto Sc-Ade plates containing 5 μg/ml adenine. Strains used for the rDNA recombination assay were all *RAD5* except KRY 1973. The recombination frequency was measured for KRY 1973 by comparing the frequency with appropriate controls by growing them on glucose (no expression of Tof2) and galactose (overexpression of Tof2). KRY 1966 and KRY 1973 were obtained by crossing W6598 (moderate overexpression of Tof2) and W6599 (higher levels of Tof2) (42) with KRY 486, respectively.

Strains were grown in appropriate medium and plated onto Sc-Ade plates containing 5 μg/ml adenine. Statistical significance was calculated using the Mann-Whitney test.

### Chromatin immunoprecipitation.

At least three independent chromatin immunoprecipitation (ChIP) experiments were done for each strain as described previously (44). Briefly, 1.5 to 1.0 OD_600_ units of a 50-ml culture of cells was cross-linked with 1% formaldehyde for 15 min and then quenched with 3.4 ml of 2 M glycine for 10 min. The cells were then pelleted and washed with ice-cold Tris-buffered saline (TBS). Cells were lysed in 800 μl of ice-cold lysis buffer with protease inhibitor (0.1% deoxycholic acid, 1 mM EDTA, 50 mM HEPES-KOH [pH 7.5], 140 mM NaCl, 1% Triton X-100) by addition of an equal volume of glass beads and vortexed at maximum speed for 20 min at 4°C. Lysate was sonicated to shear the chromatin to an average length of 200 to 800 bp. Fifty microliters of sample was taken in a fresh tube and used as input DNA. Samples were incubated overnight with primary antibody at 4°C with constant rotation. Twenty microliters of protein A-DynaMag beads was added to the chromatin-antibody mixture and incubated for 2 h at 4°C with constant rotation. Protein A-DynaMag beads were washed with 1 ml each of lysis buffer, lysis 500 buffer, LiCl-detergent solution, and TBS buffer. Chromatin immunoprecipitate was eluted first with 100 μl of 1% SDS in Tris-EDTA (TE) and then with 150 μl of 0.67% SDS in TE buffer by incubation at 65°C for 10 min. DNA from bound and unbound chromatin (input sample) was purified by phenol-chloroform-isoamyl alcohol extraction and ethanol precipitation after RNase and proteinase K digestion. DNA from ChIP experiments was analyzed by real-time PCR using Sybr green master mix on a Quant Studio 3 real-time PCR machine. The primers used are located in the NTS1 and NTS2 regions of the rDNA locus as described in reference 45. *NTS1 P1* corresponds to the RFB region at the rDNA (Fig. 1a). The enrichment was calculated at the respective loci and plotted as percent input. An average from three or six independent trials was plotted with standard error of the mean (SEM). Statistical significance between wild-type and *siz2Δ* strains across the three primer sets was calculated using two-way analysis of variance (ANOVA).

### Protein extract preparation and Western blotting.

Total protein was isolated using the trichloroacetic acid (TCA) precipitation method. Overnight culture was taken, and cells were harvested by centrifuging them for 2 min at 4,000 rpm. The cell pellet was resuspended in 200 μl of 20% TCA, and a 200-μl volume of glass beads was added and vortexed for 1 min at high speed at room temperature (RT). Cell suspension was transferred into a new 1.5-ml centrifuge tube. Glass beads were washed twice with 200 μl of 5% TCA, and the washes were added to the previous suspension. Cell pellet was collected by centrifugation at 3,000 rpm for 10 min and resuspended in 200 μl of 1× Laemmli buffer. Twenty to 30 μl of 1 M Tris base (no pH adjustment) was added until it turned blue. The sample was boiled for 5 min and centrifuged again at 3,000 rpm for 5 min. Protein sample was transferred to a new microcentrifuge tube, and the pellet was discarded. Protein was run on 8% SDS-PAGE gels and transferred to a polyvinylidene difluoride (PVDF) membrane. Anti-Myc staining was done with Ab9106 (Abcam) to detect Myc-tagged Tof2, Csm1, and Fob1, and anti-Sir2 from Santa Cruz Biotechnology was used to detect Sir2. The total protein obtained after the TCA precipitation method was diluted before loading onto the gel such that the difference in protein in a wild-type strain and a *siz2* mutant strain can be compared across the various dilutions. Statistical significance for the quantification data between wild-type and *siz2Δ* strains was determined using Mann-Whitney test with at least three or more independent biological replicates.

### Data availability.

CHiP data have been deposited at https://doi.org/10.6084/m9.figshare.10296542.v2.
